# Nurses’ experiences of compassionate care in the palliative pathway

**DOI:** 10.1111/jocn.15528

**Published:** 2020-10-25

**Authors:** Anett Skorpen Tarberg, Bodil J. Landstad, Torstein Hole, Morten Thronæs, Marit Kvangarsnes

**Affiliations:** ^1^ Medical department Møre and Romsdal Hospital Trust Ålesund Norway; ^2^ Department of Clinical and Molecular Medicine Faculty of Medicine and Health Science European Palliative Care Centre (PRC) Norwegian University of science and Technology (NTNU Trondheim Norway; ^3^ Department of Health Sciences Mid Sweden University Östersund Sweden; ^4^ Levanger Hospital Nord‐Trøndelag Hospital Trust Levanger Norway; ^5^ Faculty of Medicine and Health Sciences NTNU ‐ Norwegian University of Science and Technology Trondheim Norway; ^6^ Cancer Clinic St. Olav Hospital Trondheim University Hospital Trondheim Norway; ^7^ Department of Health Sciences in Ålesund Faculty of Medicine and Health Sciences NTNU – Norwegian University of Science and Technology Ålesund Norway; ^8^ Department of Research and Innovation Møre and Romsdal Hospital Trust Ålesund Norway

**Keywords:** cancer, compassion, nurses, palliative pathway, primary care

## Abstract

**Aims and objectives:**

The aim was to explore how nurses experience compassionate care for patients with cancer and family caregivers in different phases of the palliative pathway.

**Background:**

Compassion is fundamental to palliative care and viewed as a cornerstone of high‐quality care provision. Healthcare authorities emphasize that patients should have the opportunity to stay at home for as long as possible. There are, however, care deficiencies in the palliative pathway.

**Design:**

This study employed a qualitative design using focus groups and a hermeneutic approach.

**Methods:**

Four focus groups with three to seven female nurses in each group were conducted in Mid‐Norway in 2018. Nurses’ ages ranged from 28–60 years (mean age = 45 years), and they were recruited through purposive sampling (*N* = 21). Compassionate care was chosen as the theoretical framework. Reporting followed the COREQ guidelines.

**Results:**

Three themes expressing compassionate care related to different phases of the pathway were identified: (a) information and dialogue, (b) creating a space for dying and (c) family caregivers’ acceptance of death.

**Conclusions:**

This study showed that it was crucial to create a *space for dying*, characterized by trust, collaboration, good relationships, empathy, attention, silence, caution, slowness, symptom relief and the absence of noise and conflict.

**Relevance to clinical practice:**

The quality of compassion possessed by individual practitioners, as well as the overall design of the healthcare system, must be considered when creating compassionate care for patients and their family caregivers. Nursing educators and health authorities should pay attention to the development of compassion in education and practice. Further research should highlight patients’ and family caregivers’ experiences of compassionate care and determine how healthcare systems can support compassionate care.

**What does this paper contribute to the wider global clinical community?**

- It provides insight into nurses’ role in compassionate care in different phases of the palliative pathway.
- It highlights the importance of early engagement with family caregiver as a key element of compassionate care.
- Nurses play a crucial role in creating *a space for dying w*hich is important for patients’ and their family members’ preparation for death.

## 1 INTRODUCTION

Compassion is fundamental to palliative care and can create an environment of safety for patients and family caregivers. Compassionate care is built on trust and good relationships between the patient, the family and healthcare personnel (Brito‐Pons & Librada‐Flores, 2018; Larkin, 2016).

There are various definitions of compassionate care (Crawford et al., 2014; Feo et al., 2018; Strauss et al., 2016). In this study, we followed a broad description of compassion as involving an awareness of, or a sensitivity to, the pain or suffering of others that results in taking verbal, nonverbal or physical action to remove, reduce or alleviate the impact of such affliction (Gilbert, 2013). This description is relevant because research has shown that patients and their family caregivers experience deficiencies in palliative care provision (McEwen et al., 2018; Røen et al., 2018; Tarberg et al., 2019). A Norwegian study found that family caregivers experienced limited involvement, a lack of preparation for the dying phase, and unsystematic follow‐up after death (Tarberg et al., 2019). An Australian study showed a gap between guidelines and family caregivers’ experiences of emotional and psychological support in palliative care (Aoun et al., 2017).

The integration of palliative care with oncology is recommended; however, this has been insufficiently addressed in healthcare systems (Kaasa et al., 2018). Six main elements of patient‐centred care are highlighted: (1) respect for patients’ values, preferences, and expressed needs; (2) coordination and integration of care; (3) information, communication, and education; (4) physical comfort‐relief of bothersome symptoms; (5) emotional support‐relief of fear and anxiety; and (6) involvement of family and friends (Kaasa et al., 2018). Nurses can play a key role in integrating palliative care and oncology by providing compassionate care (Brito‐Pons & Librada‐Flores, 2018).

In this study, we explore nurses’ experiences of compassionate care for patients and family caregivers in the palliative pathway. Nurses work closely with patients and family caregivers and are therefore a relevant population in which to explore compassion.

## 2 BACKGROUND

One recent study, which included participants from 15 countries, explored nurses’ understanding of compassion (Papadopoulos et al., 2017). Nurses reported that sociopolitical structures constrained and influenced their provision of care. Lack of time was also identified as an obstacle for the provision of compassionate care. Five components were identified as comprising compassion: (1) investing time in the nurse–patient relationship, (2) presence, (3) going the extra mile, (4) personalization and (5) advocacy (Papadopoulos et al., 2017).

Compassion requires action (Larkin, 2016). True compassion is expressed through the highest level of clinical practice, which addresses the totality of symptom burden and complex needs. Compassion implies a sense of coherence, nurses being able to communicate a compassionate essence, based on knowledge, proactivity and interconnectedness in the delivery of nursing. Compassion is not just about individual responses, but rather about how nurses are enabled by the system to sustain and support themselves in the complexity of palliative care (Larkin, 2016).

To support the patient in the process of dying, previous researchers have identified some key elements deemed important by community nurses: symptom control, patient choice, honesty, spirituality, interprofessional relationships, organization and the provision of seamless care (Griggs, 2010). Building trust and knowledge with patients and their families is valuable during end‐of‐life care. Building trust depends on nurses’ availability (Stajduhar et al., 2011). Compassionate care facilitation includes the personal and relational characteristics of the primary care nurse, the organizational framework and an individually tailored care system. Barriers to compassion include personal challenges, relational challenges, system challenges and maladaptive responses (Singh et al., 2018).

Nurses have a coordinating role between patients, families and other health professionals, which is also challenging (Sekse et al., 2018). Wilson et al. (2014) reported that primary care nurses have noted that family dynamics impact on complex and difficult situations. The family, patient and nurses may all be at different stages in the acceptance of death. Further, conflict may arise when patients conceal information about their medication or misunderstand and feel suspicious around its use (Lund et al., 2015; Wilson et al., 2014).

Many of the definitions of compassionate care are general and do not consider that compassionate care will have different expressions in different contexts for different patients and situations. A discursive paper from New Zealand presented a bi‐cultural approach to providing compassionate care during end‐of‐life care (Robinson et al., 2019). The Kapakapa Manawa Framework was developed by drawing on empirical research that captured the experiences of palliative care in hospitals from the perspectives of bereaved families (Dewar & Nolan, 2013; Durie, 1985; Gott et al., 2019). The researchers extended the framework to encompass Māori values of compassion during end‐of‐life care. This model differs from others by noting how compassion should be integrated into nursing practice by referring explicitly to compassion as a verb. The model considers patients’ cultural background in care provision and the family members involved, which may be used to support the implementation of the relational component of ‘Fundamentals of Care’ (Robinson et al., 2019). Knowing enough about patients and developing trust is an important element in this framework. Conceptualizing compassion as an action may be used as a platform on which to develop meaningful relationships (Robinson et al., 2019).

This framework outlines four values, which optimize compassionate nursing in the palliative pathway: (1) relationships that express care, (2) the process of establishing good relationships, (3) the use of contextualized knowledge and (4) a reciprocal process of mutual respect between people. This model refers to a Māori concept that relates to the process of establishing relationships and nurturing ongoing connections through effective inter‐relational caring. This understanding of compassion brings the nurse and the patient closer together and provides a better understanding of the patient as a person (Robinson et al., 2019).

In our study, we explored compassionate care in the Norwegian context. In Norway, 13 per cent of the population died at home in 2018 (The Norwegian Institute of Public Health, 2019). The health authorities have recommended that, as more patients choose to stay longer at home, they should have the opportunity to die at home (Norwegian Ministry of Health & Care Service, 2020). Targeted measures have been designed to give everyone a dignified end of life in line with their needs and wishes. Expertise on palliation, resources and cooperation is necessary to enable nurses to fulfil these aims (Kaasa et al., 2018).

In this study, we understand compassionate care as consisting of three dimensions: noticing, feeling and responding. In addition, we consider compassionate care as an overall design of healthcare organizations (Blomberg et al., 2016; Crawford et al., 2014; Gilbert, 2013; Kanov et al., 2004; Larkin, 2016). We have divided the palliative pathway in three different parts: the first phase is defined as the first days following the diagnosis of an incurable disease, the second phase is the middle part of the incurable disease, and the third phase, also termed as the terminal phase, constitutes the last weeks and days before death (Tarberg et al., 2019). The aim was to explore how nurses experience compassionate care for patients with cancer and family caregivers in different phases of the palliative pathway.

## 3 METHODS

### 3.1 Design

The study employed a qualitative design with a hermeneutic approach (Gadamer, 1989; Patton, 2015). Focus groups were chosen to explore nurses’ experiences through discussions with other participants with whom they had something in common – in order to promote self‐disclosure (Brinkmann & Kvale, 2015; Krueger & Casey, 2015; Malpas & Gannder, 2017). The Consolidated Criteria for Reporting Qualitative Checklist were followed, see File S1 (Tong et al., 2007).

### 3.2 Sampling

Informants were chosen by means of purposive sampling (*N* = 21) (Brinkmann & Kvale, 2015; Krueger & Casey, 2015). Four focus groups with three to seven female nurses in each group participated in the study. Nurses’ ages ranged from 28‐60 years (mean age = 45 years). Nurses from primary care facilities and from nursing homes were recruited because they had experiences in different phases of palliative care. Nurses from urban and rural areas were also included to increase data variation. Participants worked in different municipalities in Mid‐Norway with 2000 to 43,000 inhabitants. Inclusion criteria were nurses who had worked in palliative care for more than three years and who could speak fluent Norwegian. Administrative nurses were excluded. Participants’ demographic characteristics are shown in Table 1.

**TABLE 1:** Characteristics of study participants

	Participants (total *n* = 21)
Experience in home care	7
Experience in a community institution	9
Experience in home care and community institution	5
Female	21
Male	0
Registered nurse	21
Palliative nurse	2
Oncology nurse	6
<30 years	1
31–40 years	8
41–50 years	3
51–60 years	9
Nurses from urban areas	10
Nurses from rural areas	11

### 3.3 Data collection

Nurses were recruited face‐to‐face by contact persons in the municipalities. A question route with open‐ended questions was developed based on the study aim and earlier research (Crawford et al., 2014; Krueger & Casey, 2015; Tarberg et al., 2019). The questions were related to how nurses had experienced compassionate care in different phases of the palliative pathway: namely the first, the second and the third phase. The question route was as follows:

1. Can you tell me how you experience palliative care?
2. What is important when communicating with patients and family caregivers in different phases of the palliative pathway?
3. How do you wish to collaborate with family caregivers throughout the pathway?
4. What is important about the nature of the care offered in different phases of the palliative pathway?
5. What challenges and ethical dilemmas did you experience?
6. Is there something else you want to add?

The first author was a moderator and the second author was an assistant – taking field notes and summarizing what nurses said at the end of the interviews. The focus groups, conducted in Norwegian, lasted between 60 and 90 min. They were audio‐recorded and transcribed verbatim shortly thereafter by the first author (Krueger & Casey, 2015; Polit & Beck, 2012).

The interviews provided rich descriptions of nurses’ perception of compassionate care in different phases of the palliative pathway. Data were collected in 2018, until no substantially new information was obtained from the last group. We considered that the data were saturated regarding all the preliminary themes. Saturation was discussed between the researchers after the interviews. Data collection and analysis went hand‐in‐hand (Patton, 2015). The moderator let the discussion flow naturally between participants, that is they were given the opportunity to speak openly and to participate in the focused discussion. (Krueger & Casey, 2015).

### 3.4 Data analyses

We used compassionate care as a theoretical framework when interviewing the nurses. All the authors read the interviews to gain a holistic impression of the data (Brinkmann & Kvale, 2015). The first author coded the interviews related to compassionate care in the first, second and third palliative phase. The first author has worked as an oncology nurse in primary care for 10 years. Leaning on a hermeneutic approach, we were aware that her preunderstanding influenced data interpretation (Gadamer, 1989); therefore, all authors engaged in discussing the analyses and a new understanding was developed from group discussions (Brinkmann & Kvale, 2015). We used the hermeneutic circle, in which the meaning of the parts is determined by the global meaning. Consequently, we gained a new and deeper understanding of compassionate care in different phases of the pathway – both for patients and for their family caregivers (Gadamer, 1989). In the process of interpretation, it was important to read the interviews with empathy, that is we tried to understand the intentions behind what was said. This enriched our previous interpretations. In all our interpretations, our perceptions of the nurses’ view of compassionate care were central (Alvesson & Sköldberg, 2018). Quotations, subthemes and themes are presented in Table 2.

**TABLE 2:** Development of quotes into themes

Quotes	Subthemes	Theme
Theme (a)		
‘When we manage to establish early contact, it becomes easier to work together at the end.’	*Early involvement of primary care nurses*	
‘We need to help them create a palliative plan and clarify important aspects, try to avoid situations where decisions must be made quickly, and where patients and family caregivers may not be prepared.’	*Advance care planning*	Information and dialogue
‘It was a good process because we cooperated: palliative team, general practitioner, the nursing home and family caregivers.’	*The family caregivers as a part of the team*	
Theme (b)		
‘We have the opportunity to create a space, where patients and families can prepare for death.’	*Trust*	
‘Family caregivers sometime express: ‘You must get the patient to the nursing home, but please don`t tell him/her that the words come from us.’’	*To balance conflict of interest*	Creating a space for dying
‘There was a mother with small children who said she hoped to recover. The nurse then replied: ‘Yes, I hope so too, but we must have an alternate plan.’’	*Emotional reciprocity*	
Theme (c)		
‘Family caregivers require explanations about the dying process, and how to meet the needs of a dying patient.’	*Common understanding of the treatment*	
‘We offer bereavement counseling four to six weeks after the death.’	*Routine of bereavement counselling after death*	Family caregivers’ acceptance of death
‘This provides an opportunity to ask questions about what occurred.’	*Communication about the process of dying*	

### 3.5 Ethical considerations

The project was undertaken according to research ethics guidelines (World Medical Association, 2013). The Regional Committee on Medical and Health Research Ethics determined that the study did not require ethical approval (no. 2016/978/REK NORD). The Data Protection Official for Research approved this study (no. 2016/960‐25). All nurses were given oral and written information that they could withdraw whenever they wanted. Informed written consent was obtained by all nurses at the start of the interviews. All data were anonymized. The informants were colleagues, and we were conscious of presenting the interviews in a respectful manner (Brinkmann & Kvale, 2015).

### 3.6 Rigour

Decisions were carefully described to enhance the transparency of this study (Polit & Beck, 2012) and to enable readers to evaluate the research process. Two researchers conducted the interviews, and both were experienced in performing qualitative interviews. The theoretical framework was carefully described to increase data interpretation validity (Patton, 2015). A coding tree and various stages in the analysis were described to enhance reliability in the analysis. All authors participated in discussions about data interpretation (Tong et al., 2007). Participants’ quotations were presented to illustrate the themes (Table 2).

## 4 RESULTS

Twenty‐one nurses working in palliative care shared their experiences of compassionate care for patients and family caregivers in the palliative pathway. Three themes were identified: (a) information and dialogue in the first phase, (b) creating a space for dying and (c) family caregivers’ acceptance of death. The first and the second theme relate to compassionate care for patients and family caregivers in the first and second phase of the palliative pathway, respectively. The third theme relates to family caregivers’ acceptance of death in the second and third phase.

### 4.1 Information and dialogue

Nurses emphasized the importance of early contact in order to provide information about what services they could offer. They often had little contact with patients and family caregivers in the first phase of the pathway. Nurses conveyed that patients and family caregivers felt shock and sadness in this first phase, and often they were not ready to meet oncology nurses from primary care. The nurses thought that this might contribute to a delayed provision of health services. An explanation provided by them was that patients and family caregivers might not have sufficient knowledge or experience to understand the importance of early involvement with health personnel: ‘*When we manage to establish early contact*, *it becomes easier to work together at the end*’. Hence, the nurses highlighted the importance of dialogue between patients, family caregivers and healthcare personnel through the course of the disease, and noted that early involvement increased their ability to provide compassionate care. Interdisciplinary collaboration between specialist healthcare services and primary healthcare was considered important to improve compassionate care.

The nurses emphasized that physicians and nurses in the hospitals had a key role in communicating the importance of early involvement in primary health services. They considered it vital to plan the palliative pathway together with the patients and family caregivers before the patient had reached the third and terminal phase. The need for advance care planning was described: ‘*We need to help them create a palliative plan and to clarify important aspects*, *try to avoid situations where decisions must be made quickly*, *and where patients and family caregivers may not be prepared’*. The nurses indicated that a palliative plan should provide patient‐centred care and carry out the patient's wishes. They experienced that advance care planning led to useful information being conveyed to patients and family caregivers, created a sense of security and prepared patients and family caregivers for what was to come.

Nurses discussed the value of including family caregivers as part of the team. ‘*It was a good process because we cooperated*: *palliative team*, *general practitioner*, *the nursing home and family caregivers*’. Close collaboration between family caregivers, primary care providers and healthcare specialists made it possible to fulfil patients’ wishes to die at home. Collaboration was seen as an important element of compassionate care.

### 4.2 Creating a space for dying

The second phase needed to be a quiet period in which patients and family caregivers were provided with security, predictability and clarification. Nurses were engaged and emotionally affected when they talked about this topic. They emphasized the importance of creating a space for dying, and that there were better facilities than hospitals in which to create this space: ‘*We have the opportunity to create a space*, *where patients and families can prepare for death*’. According to the nurses, a space for dying was characterized by trust, collaboration, good relationships, empathy, attention, silence, caution, slowness, symptom relief and the absence of noise and conflict. Nurses perceived that patients and families had best experienced compassionate care in primary healthcare at home and in nursing homes.

Challenges in the interactions with patients and family caregivers were a topic in the focus groups. Balancing a conflict of interest between family members could be challenging and could prevent adequate planning for the impending death. This could hinder the process of ‘creating a space for dying’. Nurses expressed that patients and family caregivers, as well could have different needs: *‘Family caregivers sometimes express*, *“You must get the patient to the nursing home*; *but please don't tell him*/*her that the words come from us*.*”’* In such a situation, just whose interests should be prioritized, becomes an ethical dilemma for health personnel. Nurses had to be aware of patients’ and family caregivers’ mental and physical needs. The importance of trust and good interpersonal relationships in providing compassionate care was crucial.

Communication skills were also an essential competence with regard to providing compassionate care. Nurses described how they tried to prepare patients and families for the last days and death. They indicated that certain patients could not relate to their impending death: *‘There was a mother with small children who said she hoped to recover*. *The nurse then replied*, *“yes*, *I hope so too*; *but we must have an alternate plan*.*”’* This way of responding to the patient illustrates that the nurse is listening to the patient in a way that conveys both hope and realism. Communicating in an empathic way is an important part of compassionate care.

Another recurrent topic was the importance of nurses having expertise in symptom relief. Nurses experienced that there was a lack of collaboration between physicians and nurses. This could result in patients not receiving adequate medication in time. The nurses argued for the importance of interprofessional collaboration with regard to the provision of symptom‐relieving medication in the third phase. This allowed for combined planning and the anticipation of possible difficulties, at a system level. It also required that professionals find new ways of collaborating with each other.

### 4.3 Family caregivers’ acceptance of death

The nurses experienced the last phase as difficult. Dilemmas arose when healthcare professionals and family caregivers had a different understanding of treatment choices; for instance, if family caregivers wanted health personnel to provide treatment and the patient did not want it. The need for information to family caregivers about palliative treatment was highlighted, especially relating to fluid and nutrition: ‘*Family caregivers require explanations about the death process*, *and how to meet the needs of a dying patient*’. Nurses had experienced that family caregivers could become despairing and angry when the patient could not eat and drink in the third phase. They had often experienced that treatment limits had not been made clear in advance. Hence, a common understanding between healthcare personnel and family caregivers was important.

After a patient's death, nurses had bereavement routines to follow, in which contact was offered to grieving family members: ‘*We offer bereavement counseling*, *four to six weeks after the death*’. Nurses in the focus groups vehemently discussed communication with the bereaved. The routines seemed, however, to differ both between municipalities and within municipalities. In some municipalities, nurses offered calls only to the bereaved family of patients who had died of cancer.

Nurses experienced that the bereaved had different needs, and some nurses expressed that it was especially important for the bereaved to meet the health professionals who had been present when the patient died. A nurse expressed it like this, *‘This provides an opportunity to ask questions about what occurred’*. Some of the bereaved needed several conversations to get over the loss of their beloved ones. Nurses thought that almost everyone would benefit from a conversation with healthcare personnel after a patient had died. To help the bereaved to get over their loss was an important part of compassionate care.

## 5 DISCUSSION

The focus groups provided rich data and gave a new understanding of the meaning of compassionate care in different phases in the palliative pathway. The analyses have revealed that compassionate care is contextual. Information and dialogue with patients and family caregivers was crucial in the first phase. In the second phase, the nurses highlighted the importance of creating a space for dying. In the third phase, family caregivers’ acceptance of patients’ death was important.

Information and dialogue with patients and family caregivers early in the pathway was an important finding in this study. Earlier research has indicated that building trust, knowledge and good relationships are important in end‐of‐life care (Robinson et al., 2019; Stajduhar et al., 2011). This is in accordance with the results presented in this study. The nurses advised that family caregivers should be seen as part of the team around the patient.

Our study show the significance of advance care planning, which involves patients and family caregivers in the process. The goal of advance care planning is to ensure that medical care is consistent with patients’ and family caregivers’ values, goals and preferences (Kaasa et al., 2018). Nurses experienced that advance care planning gave patients and family caregivers a sense of security and prepared them for future challenges. In this, they mirror the findings of Pfaff and Markaki (2017), who, in an integrative review, highlighted the significance of collaborative and patient‐ and family‐centred care.

Nurses expressed that creating a space for dying was crucial for patients and family caregivers. The significance of creating this space has not been highlighted in previous research about compassionate care in the palliative pathway. Based on the findings in this study, it is urged that the provision of primary health care for the dying, whether at home or a nursing home, is provided with facilities, and a philosophy, which facilitates a compassionate culture for both patient and family caregiver. Larkin (2016) has argued that compassion is not just about individual responses, but rather about how practitioners are able to sustain and support themselves in the complex field of palliative care.

The importance of creating a space for dying demands that nursing managers and policymakers prioritize resources for healthcare personnel to assist them in shaping a compassionate culture (Crawford et al., 2014; Martinsen & Kjerland, 2006). Our findings support the understanding that the organization and design of services are important in compassionate care.

Family conflicts, different stages of accepting death within the family and denying families a role in decision‐making are obstacles to creating compassionate care (Lund et al., 2015; Wilson et al., 2014). In this study, nurses experienced that they played a significant role by being active in creating a space for dying. We consider this space as important for patients and families in accepting and preparing for death.

Further, a lack of interdisciplinary collaboration, specifically, physicians’ failure to prescribe sufficient pain relief medication were problematic. This may be an obstacle to patients receiving symptom relief in the third and terminal phase. Griggs (2010) describes symptom control as a key element in supporting patients’ process of dying. International recommendations (Kaasa et al., 2018) have also highlighted the importance of physical comfort‐relief of bothersome symptoms. Nurses experienced that they play a crucial role in collaboration with physicians to ensure that a patient receives effective pain relief. The supply of care and medication needs to be well‐organized, so that dying patients get symptom relief also in weekends and holidays.

### 5.1 Strengths and Limitations

This study illustrated compassionate care in the palliative pathway from the perspective of nurses and not from the experiences of patients and family caregivers. The study provided rich data, which we believe offers new insight into compassionate care in various phases of the palliative pathway. In the future, it is suggested that compassionate care in the palliative pathway be studied from the perspectives of patients, family caregivers and physicians, to enable us to develop a more holistic understanding.

Compassionate care was chosen as the theoretical framework (Blomberg et al., 2016; Brito‐Pons & Librada‐Flores, 2018; Crawford et al., 2014; Robinson et al., 2019). This framework had an impact on how the data were collected and interpreted. A hermeneutic approach assumes that the findings are an interpretation based on a theoretical framework and should be interpreted in a cultural and historical context (Patton, 2015). This background is important in interpreting compassionate care expressed by nurses in the Norwegian context. In Norwegian, there are no expressions that are synonymous with ‘compassionate care’. It was therefore important to have a theoretical framework when studying this phenomenon in the Norwegian context. There is thus a need to develop concepts in Norwegian which communicate the content of compassionate care in the community of practice in Norway.

Although this study was conducted in Norway, the findings may be generalized to other countries with similar health services (Polit & Beck, 2012). The theoretical framework (Blomberg et al., 2016; Crawford et al., 2014; Gilbert, 2013; Kanov et al., 2004; Larkin, 2016) was important in revealing key elements of compassionate care at various stages of the pathway. In the interviews, we used Norwegian terms that corresponded with terms and concepts in the English theoretical framework of compassionate care. The study provides new insights of international relevance because the findings reveal existential and general challenges related to caring in the palliative pathway.

The first author has been working as an oncology nurse in primary health care for many years, and she has experience in the concepts discussed in this study. However, all the authors collaborated in the data interpretation to develop a new understanding and to ensure a holistic perspective (Gadamer, 1989; Patton, 2015).

## 6 CONCLUSION

Compassionate care is different in the three phases of the pathway, and the nurses should take an active role in creating compassionate care throughout the pathway. It is crucial to create a space for dying. Trust, collaboration, good relationships, empathy, attention, silence, caution, slowness, symptom relief and absence of noise and conflicts characterize compassionate care when crating a space for dying. It is likely that the findings can provide insight into caring in the palliative pathway for patient groups with other chronic diseases.

## 7 RELEVANCE TO CLINICAL PRACTICE

Nurses should involve family caregivers as a part of the team around the patient in the first phase of the palliative pathway. It is important that nurses spend time building trust. Nurses should take a coordinating role in creating a space for dying. Managers and policymakers should prioritize resources for healthcare services in shaping compassionate culture. Healthcare personnel should offer bereavement counselling in a systematic way. Compassionate care in the different phases of the palliative pathway should be addressed in nursing education and further research. In the future, investigations of patients, family caregivers, physicians and policymakers perspectives of compassionate care could present us with a more holistic understanding.

## CONFLICT OF INTEREST

There are no conflict of interest to declare.

## AUTHOR CONTRIBUTIONS

All authors made substantial contributions to conception and design, or acquisition of data, or analysis and interpretation of data. AST and MK conducted the interviews, AST transcribed them verbatim. All authors were involved in drafting the manuscript or revising it critically for important intellectual content. All authors have given final approval of the version to be published, participated sufficiently in the work to take public responsibility for appropriate portions of the content. All authors agreed to be accountable for all aspects of the work in ensuring that questions related to the accuracy or integrity of any part of the work are appropriately investigated and resolved.

## Supporting information

File S1

## References

[jocn15528-bib-0001] Alvesson, M. , & Sköldberg, K. (2018). Reflexive methodology: New vistas for qualitative research (3rd ed.). Los Angeles, California: SAGE.

[jocn15528-bib-0002] Aoun, S. M. , Rumbold, B. , Howting, D. , Bolleter, A. , & Breen, L. J. (2017). Bereavement support for family caregivers: The gap between guidelines and practice in palliative care. PLoS One, 12(10), e0184750. 28977013 10.1371/journal.pone.0184750 PMC5627900

[jocn15528-bib-0003] Blomberg, K. , Griffiths, P. , Wengström, Y. , May, C. , & Bridges, J. (2016). Interventions for compassionate nursing care: A systematic review. International Journal of Nursing Studies, 62, 137‐155. 27494429 10.1016/j.ijnurstu.2016.07.009

[jocn15528-bib-0004] Brinkmann, S. , & Kvale, S. (2015). InterViews: Learning the craft of qualitative research interviewing (3rd ed.). Thousand Oaks, California: SAGE.

[jocn15528-bib-0005] Brito‐Pons, G. , & Librada‐Flores, S. (2018). Compassion in palliative care: A review. Current Opinion in Supportive Palliative Care, 12(4), 472‐479. 30300152 10.1097/SPC.0000000000000393

[jocn15528-bib-0006] Crawford, P. , Brown, B. , Kvangarsnes, M. , & Gilbert, P. (2014). The design of compassionate care. Journal of Clinical Nursing, 23(23–24), 3589‐3599. 24837168 10.1111/jocn.12632

[jocn15528-bib-0007] Dewar, B. , & Nolan, M. (2013). Caring about caring: Developing a model to implement compassionate relationship centred care in an older people care setting. International Journal of Nursing Studies, 50(9), 1247‐1258. 23427893 10.1016/j.ijnurstu.2013.01.008

[jocn15528-bib-0008] Durie, M. H. (1985). A Maori perspective of health. Social Science & Medicine, 20(5), 483‐486. 3992288 10.1016/0277-9536(85)90363-6

[jocn15528-bib-0009] Feo, R. , Kitson, A. , & Conroy, T. (2018). How fundamental aspects of nursing care are defined in the literature: A scoping review. Journal of Clinical Nursing, 27(11–12), 2189‐2229. 29514402 10.1111/jocn.14313

[jocn15528-bib-0010] Gadamer, H.‐G. (1989). Truth and method (2nd, rev. ed.). New York: Continuum.

[jocn15528-bib-0011] Gilbert, P. (2013). The compassionate mind: A new approach to life's challenges (Rev. ed.). London: Robinson.

[jocn15528-bib-0012] Gott, M. , Robinson, J. , Moeke‐Maxwell, T. , Black, S. , Williams, L. , Wharemate, R. , & Wiles, J. (2019). ‘It was peaceful, it was beautiful’: A qualitative study of family understandings of good end‐of‐life care in hospital for people dying in advanced age. Palliative Medicine, 33(7), 793‐801. 31027476 10.1177/0269216319843026

[jocn15528-bib-0013] Griggs, C. (2010). Community nurses’ perceptions of a good death: a qualitative exploratory study. International Journal of Palliative Nursing, 16(3), 140‐149. 10.12968/ijpn.2010.16.3.47326 20357707

[jocn15528-bib-0014] Kaasa, S. , Loge, J. H. , Aapro, M. , Albreht, T. , Anderson, R. , Bruera, E. , Brunelli, C. , Caraceni, A. , Cervantes, A. , Currow, D. C. , Deliens, L. , Fallon, M. , Gómez‐Batiste, X. , Grotmol, K. S. , Hannon, B. , Haugen, D. F. , Higginson, I. J. , Hjermstad, M. J. , Hui, D. , … Lundeby, T. (2018). Integration of oncology and palliative care: A Lancet Oncology Commission. The Lancet Oncology, 19(11), e588‐e653. 30344075 10.1016/S1470-2045(18)30415-7

[jocn15528-bib-0015] Kanov, J. M. , Maitlis, S. , Worline, M. C. , Dutton, J. E. , Frost, P. J. , & Lilius, J. M. (2004). Compassion in organizational life. American Behavioral Scientist, 47(6), 808‐827.

[jocn15528-bib-0016] Krueger, R. A. , & Casey, M. A. (2015). Focus groups: a practical guide for applied research (5th ed.). Los Angeles, California: SAGE.

[jocn15528-bib-0017] Larkin P. J. (Ed.) (2016). Compassion: The essence of palliative and end‐of‐life care. Oxford: Oxford University Press.

[jocn15528-bib-0018] Lund, L. , Ross, L. , Petersen, M. A. , & Groenvold, M. (2015). The interaction between informal cancer caregivers and health care professionals: A survey of caregivers’ experiences of problems and unmet needs. Supportive Care in Cancer, 23(6), 1719‐1733. 25432867 10.1007/s00520-014-2529-0

[jocn15528-bib-0019] Malpas J., & Gander H.‐H. (Eds.) (2017). The Routledge companion to hermeneutics. London: Routledge.

[jocn15528-bib-0020] Martinsen, K. , & Kjerland, L. E. (2006). Care and vulnerability. Oslo: Akribe.

[jocn15528-bib-0021] McEwen, R. , Asada, Y. , Burge, F. , & Lawson, B. (2018). Associations Between Home Death and the Use and Type of Care at Home. Journal of Palliative Care, 33(1), 26‐31. 29332502 10.1177/0825859717751933

[jocn15528-bib-0022] Norwegian Ministry of Health and Care Service (2020). Lindrende behandling og omsorg [Palliative treatment and care] (Meld. St. 24 (2019–2020)). https://www.regjeringen.no/no/dokumenter/meld.‐st.‐24‐20192020/id2700942/

[jocn15528-bib-0023] Papadopoulos, I. , Taylor, G. , Ali, S. , Aagard, M. , Akman, O. , Alpers, L.‐M. , Apostolara, P. , Biglete‐Pangilinan, S. , Biles, J. , García, Á. M. , González‐Gil, T. , Koulouglioti, C. , Kouta, C. , Krepinska, R. , Kumar, B. N. , Lesińska‐Sawicka, M. , Diaz, A. L. L. , Malliarou, M. , Nagórska, M. , … Zorba, A. (2017). Exploring nurses’ meaning and experiences of compassion: An international online survey involving 15 countries. Journal of Transcultural Nursing, 28(3), 286‐295. 26718861 10.1177/1043659615624740

[jocn15528-bib-0024] Patton, M. Q. (2015). Qualitative research & evaluation methods: Integrating theory and practice (4th ed.). Sage.

[jocn15528-bib-0025] Pfaff, K. , & Markaki, A. (2017). Compassionate collaborative care: An integrative review of quality indicators in end‐of‐life care. BMC Palliative Care, 16(1), 65. 29191185 10.1186/s12904-017-0246-4 PMC5709969

[jocn15528-bib-0026] Polit, D. F. , & Beck, C. T. (2012). Nursing research: Generating and assessing evidence for nursing practice (9th ed.). Philadelphia, Pennsylvania: Wolters Kluwer Health.

[jocn15528-bib-0027] Robinson, J. , Moeke‐Maxell, T. , Parr, J. , Slark, J. , Black, S. , Williams, L. , & Gott, M. (2019). Optimising compassionate nursing care at the end of life in hospital settings. Journal of Clinical Nursing, 29(11–12), 1788‐1796. 31495001 10.1111/jocn.15050

[jocn15528-bib-0028] Røen, I. , Stifoss‐Hanssen, H. , Grande, G. , Brenne, A.‐T. , Kaasa, S. , Sand, K. , & Knudsen, A. K. (2018). Resilience for family carers of advanced cancer patients—how can health care providers contribute? A qualitative interview study with carers. Palliative Medicine, 32(8), 1410‐1418. 29852808 10.1177/0269216318777656

[jocn15528-bib-0029] Sekse, R. J. T. , Hunskår, I. , & Ellingsen, S. (2018). The nurse's role in palliative care: A qualitative meta‐synthesis. Journal of Clinical Nursing, 27(1–2), e21‐e38. 28695651 10.1111/jocn.13912

[jocn15528-bib-0030] Singh, P. , Raffin‐Bouchal, S. , McClement, S. , Hack, T. F. , Stajduhar, K. , Hagen, N. A. , Sinnarajah, A. , Chochinov, H. M. , & Sinclair, S. (2018). Healthcare providers’ perspectives on perceived barriers and facilitators of compassion: Results from a grounded theory study. Journal of Clinical Nursing, 27(9–10), 2083‐2097. 29575539 10.1111/jocn.14357

[jocn15528-bib-0031] Stajduhar, K. I. , Funk, L. M. , Roberts, D. , Cloutier‐Fisher, D. , McLeod, B. , Wilkinson, C. , & Purkis, M. E. (2011). Articulating the role of relationships in access to home care nursing at the end of life. Qualitative Health Research, 21(1), 117‐131. 20682963 10.1177/1049732310379114

[jocn15528-bib-0032] Strauss, C. , Taylor, B. L. , Gu, J. , Kuyken, W. , Baer, R. , Jones, F. , & Cavanagh, K. (2016). What is compassion and how can we measure it? A review of definitions and measures. Clinical Psychology Review, 47, 15‐27. 27267346 10.1016/j.cpr.2016.05.004

[jocn15528-bib-0033] Tarberg, A. S. , Kvangarsnes, M. , Hole, T. , Thronæs, M. , Madssen, T. S. , & Landstad, B. J. (2019). Silent voices: Family caregivers’ narratives of involvement in palliative care. Nursing Open, 6(4), 1446‐1454. 31660172 10.1002/nop2.344 PMC6805263

[jocn15528-bib-0034] The Norwegian Institute of Public Health (2019). Dødsårsaksregisteret – Statistikkbank [Norwegian Cause of Death Registry]. http://statistikk.fhi.no/dar/

[jocn15528-bib-0035] Tong, A. , Sainsbury, P. , & Craig, J. (2007). Consolidated criteria for reporting qualitative research (COREQ): A 32‐item checklist for interviews and focus groups. International Journal for Quality in Health Care, 19(6), 349‐357. 17872937 10.1093/intqhc/mzm042

[jocn15528-bib-0036] Wilson, C. , Griffiths, J. , Ewing, G. , Connolly, M. , & Grande, G. (2014). A qualitative exploration of district nurses’ care of patients with advanced cancer: the challenges of supporting families within the home. Cancer Nursing, 37(4), 310‐315. 23945144 10.1097/NCC.0b013e31829a9a56

[jocn15528-bib-0037] World Medical Association (2013). World Medical Association Declaration of Helsinki: Ethical Principles for Medical Research Involving Human Subjects. JAMA, 310(20), 2191‐2194. 24141714 10.1001/jama.2013.281053

